# Supplementary material for: Testing the stability of theory of mind: A longitudinal approach
Source: PLoS One. 2020 Nov 5;15(11):e0241721. doi: 10.1371/journal.pone.0241721 (PMC7644065; doi:10.1371/journal.pone.0241721)
Supplement: Attachment — Submitted filename: Reviews Responses. Plos One..docx [file pone.0241721.s002.docx]

Review Comments to the Author

Reviewer #1: The goal of the current study was to examine whether there is continuity between infants’ performance on implicit Theory of Mind (ToM) measures and their later performance on ToM tasks during the preschool years. In study 1, children were tested using false-belief and intention understanding Violation of Expectation (VOE) paradigms in infancy and traditional false-belief tasks at ages 4 and 5. There were no associations between children’s performance on the tasks in infancy and their later performance at 4 and 5. In study 2, 18-month-olds completed false-belief and knowledge inference tasks using interactive paradigms. These children were tested again at age 5 using the Wellman and Liu (2004) Theory of Mind scale and several other measures. There was an association between infants false-belief performance and their later performance on the divergent beliefs measure from the Wellman and Liu scale at age 5. No other associations were reported.

The question of whether early performance on ToM tasks is predictive of children’s later ToM understanding is an important one that is likely to be of interest to the field. However, there are several issues with the manuscript that need to be addressed.

The introduction is difficult to follow and the theoretical motivation for the study is not clearly or strongly outlined. For instance, the authors describe competing views regarding the development of ToM, but **they do not explain how these accounts interpret infants’ performance on implicit ToM tasks,** or what these different accounts would predict in terms of continuity between ToM understanding from infancy to later childhood. There is a brief mention of the “stability hypothesis” and conservative and lenient tests of this hypothesis, but this is not unpacked for the reader. In addition, hypotheses are made regarding stability of false-belief understanding across time, but what about the other aspects of ToM the authors are examining (e.g. intention understanding, knowledge inference)?

**In the revised version, we develop the rationale for more longitudinal studies that test a wide range of constructs and how such studies can address the depth of infant’s theory of mind (see p. 4). We also mention that the stability of theory of mind as assessed by infants’ ability for knowledge inference and knowledge access and belief reasoning in older children has never been tested (see p. 5-6).**

One of the key goals of the study is to examine associations between early performance on implicit and later performance on implicit and explicit ToM measures. However, these terms are never described in terms of what kinds of tasks fall under these categories. What differentiates the two kinds of tasks?

**We explain that implicit measures are based on spontaneous responses, as opposed to elicited responses. (see p. 3).**

Study 2 in particular **does not include much motivation for the measures used to examine associations in ToM from infancy to the preschool years.** The authors used two interactive ToM tasks for infants that are very different from the looking time measures used in prior studies they are aiming to replicate and those in Study 1. There is no description of why parental report measures were included in the task battery at age 5 and how those might compare to more traditional ToM tasks in terms of their relation to infants’ ToM. Finally, **there is no justification made for why individual items/measures were considered from the Wellman & Liu (2004) scale** rather than considering children’s **total scores or false-belief only scores** as has been done in prior studies (Aschersleben et al., 2008, Wellman et al., 2004, 2008, Yamaguchi et al., 2009 ). Were there specific hypotheses regarding how the infancy tasks might related to specific items on the scale?

**We now better explain why we elected to test both infants and preschoolers with a set of new tasks. Although the background literature is very limited, it was predicted that a task-specific effect would be observed with early knowledge inference predicting later knowledge access and early false belief predicting older children’s full-fledged false belief. Moreover, we clarify tha if a general form of stability is observed (infants’ concepts predicting total ToM score) it was expected to be found with both direct (scale) and indirect (CSUS) measures (see p. 22-23).**

Study 2 involved an interactive false-belief task that has been used in prior research (Buttelmann et al., 2009). As the authors note, there have been both successful and failed attempts to replicate this task. However, there are several factors that when changed from the original task are thought to contribute chance performance among infants in this paradigm (Buttelmann et al., 2018), such as whether the child is seated on the floor vs. at a table (resulting in differences in amount of time available to process events), to move toward manipulations to the boxes that make them more salient, etc. It would be useful to know whether this study was a strict replication or whether any adaptations to the task were made in the current study.

**We specify the changes made to the original task (as originally reported in Crivello et al. 2018). The main methodological change was the administration of the task at a table because of the high attrition rate due to unwillingness to move toward the Experimenter reported in Buttlelmann’s original study.**
(see p. 24)

The results sections contain redundant information. For instance, in Study 1 correlations are reported both in the text and in tables and it is not clear why additional simple regression analyses were performed (line 387), that showed very similar results to the null findings in the zero-order correlation table. Sections could be streamlined to reduce redundancy. Some of the tables (e.g., Tables 1 and 2) are somewhat cluttered. It might be useful to remove the row of p-values and instead mark significant results with asterisks or other symbols. Finally, many tests were reported in the results of this study (especially in Study 2) without giving any apriori predictions about relations between specific tasks.

**We have removed the regressions and fixed the tables. For Study 2, as mentioned above, we now make more explicit the relations that were expected across waves.**

In the discussion section, the authors posit that the reason they may not have found associations to later ToM with the VOE false-belief task is because it doesn’t actually measure understanding of false beliefs. However, they also state that the interactive helping task, where they did find associations to later ToM also doesn’t measure false-belief understanding. Shouldn’t it be the case then, that neither task would predict later ToM performance? There is also no interpretation and discussion of findings regarding the other ToM tasks in this section.

**Given that there is now no significant statistical link between later ToM and VOE or Interactive tasks, the conclusion that the task might not measure false belief applies to both cases. As for the discussion on the other ToM tasks, stability was expected as well and we have now added a section addressing this issue in each study discussion (see p. 21, 22, 35).**

Other Comments
1. The authors state that the main goal of the article was to assess the stability of false-belief understanding from infancy to early childhood. However, their study involved many ToM measures other than false-belief understanding.

**We have now clarified the scope of our study (see p. 6)**

2. In the introduction and elsewhere there are several field-specific terms that are used that some readers may be unfamiliar with (e.g. contents, location, etc.). Brief descriptions of what those terms refer to would be helpful.

**WE have now clarified these terms (see p. 6)**

3. In sections on data cleaning, the authors note that data for outliers was replaced with the closest value within normal range. Is this a common method for replacing looking-time data and if so, can a reference for this method be provided? Even if it is a common practice, it seems odd to replace data in a study examining individual differenc

**We provide a reference for this common procedure (see p. 15, 30)**

4. In the Study 1 false-belief task and goal-directed action task, please include how many seconds infants were allowed to look at the familiarization and test trials before the trial ended.

**We apologize for this omission. WE now specify that a trial could last up to 30 seconds but ended if the child looked 2 seconds away. (see p. 10-11)**

5. In the Study 1 goal-directed action task, infants saw two test trials of each kind of event (congruent, incongruent). It would be useful to have more details regarding how the proportion scores were calculated in the context of this multi-test trial task. In line with this issue, the description of the results for the goal-directed action task only mention 1 test trial. Which test trials were included in those analyses?

**We now clarify that “Looking times during the pair of congruent test trials and the pair of incongruent test trials were averaged in order to obtain the average looking time during each type of test trial. Proportion of looking time was also computed for this task by dividing the average looking time during the incongruent trials by the sum of the average congruent and average incongruent looking time.” (see p.11)**

6. In both Study 1 and Study 2, participants were excluded from the final sample at Wave 2 due to having a developmental disability or audio-visual impairments. Were their data also removed from Wave 1?

**To be included in the sample, participants had to meet inclusion criteria at either wave. (see p. 9, 24)**

7. In the results section, additional information regarding potential effects or interactions should be reported. For instance, for the VOE false-belief task in Study 1, **were there any effects of box color, side of the box, etc.?** This information should be included for any of the tasks in infancy that counterbalanced various aspects of the events and that could impact infants’ looking/performance.

**In Study 2, side and color of box was counterbalanced in the interactive FB task (see p 25). In Study 1, the location of the boxes (green box on the right side of stage for child) and direction of movement from (right to left for child) were kept constant. (see p. 10)**

8. In the description of the contents false-belief tasks it states that children had to say that they thought BandAids (or an equivalent term) were in the box before moving on with the experiment. However, in the discussion of Study 2, the fact that a large percentage of children couldn’t identify the contents of the BandAids box is given as a potential reason for why there were no associations with that task. This information seems to be in conflict.

**Given the null results after correction, this issue is less relevant. However, we point out that a large proportion of children ignored the content in Study 2, which might explain the poor performance on that task (see p. 35)**

9. The authors mention that the rich interpretation focuses on task demands playing an important role in whether children can demonstrate false-belief understanding. Given that this study involved different kinds of tasks with varying demands, it would be interesting to acknowledge this further in the discussion and reflect on how those task demands may have played a role in the current study.

**That is an excellent point. We have added a section in the discussion where we argue that the fact that we report no longitudinal links across tasks that vary in task demands indicates that such effect is robust (see p. 38)**

Reviewer #2: Testing the stability of theory of mind: A longitudinal approach
Poulin-Dubois et al.

The manuscript presents two longitudinal studies with the aim of relating implicit Theory of Mind (ToM) tasks at the age of 14/18 months (wave 1) with implicit as well as explicit ToM tasks at the age of 4/5 years (wave 2). Study 1 assessed a violation of expectation (VOE) implicit ToM task (Onishi & Baillargeon, 2005) at wave 1 and an anticipatory looking (AL) implicit plus two traditional explicit ToM tasks (Thoermer et al. 2012) at wave 2. Study 2 assessed an interactive helping ToM task (Buttelmann et al. 2009) at wave 1 and the explicit Wellman & Liu ToM scale (Wellman & Liu 2004) at wave 2. The authors found that:

(1) In study 1, most of the task performances were at chance level, except for the explicit FB location task which was below chance.
(2) There were no correlations between tasks of wave 1 and wave 2 of study 1.
(3) In study 2, task performance on the interactive helping paradigm at 18 months (wave 1) was at chance level, and only some of the explicit ToM tasks in preschool age (wave 2) had above chance performance.
(4) Most of the tasks showed no correlations between wave 1 and wave 2 in study 2, except for the interactive task at wave 1 with the diverse beliefs tasks at wave 2.
The authors interpret these results in a way that precursors of ToM might already be present as building blocks in infancy.

These two studies are definitely of big interest for the current debate about the interpretation of infant ToM findings and the question of their continuity to later ToM development in preschool-age. Despite the apparent value of these studies for the field, we have a few comments that we think should be addressed to ensure that the reported findings and their interpretation are fully valid.

Major Comments

(1) First of all, we were wondering whether the correlations reported in study 2, including the only significant correlation in the paper, were also corrected for multiple comparison. The authors only reported correction for study 1. Since a relatively large number of correlations were tested and only a single positive correlation was reported (with p = .039), this would be essential for the finding and conclusions of the study to hold.

**Thank you for noting this oversight. After such corrections, no statistically significant correlations are found. We have edited the results of Study 2 section and the General Discussion accordingly.**

(2) Given a single significant amongst mostly non-significant correlations between early infant ToM task and later explicit ToM tasks we feel that the author’s conclusion that the infant findings are a ToM precursor that provides the building blocks for later mature ToM **is too strong**. Even if this correlation survives correction for multiple comparison, we therefore suggest to phrase the conclusions more tentatively.

**Given that there are no significant correlation after correction, the general discussion has been edited accordingly. We argue that the infant tasks included in the present studies most likely measure some form of social-cognitive skills that are not predictive of later theory of mind skills.**

(3) Another problem in the interpretation of the positive correlation as well as the null-findings is that the performance of many tasks (including the interactive helping task that shows a positive correlation with later diverse belief reasoning) is at chance-level. This makes it hard to assess whether the correlated variance in these tasks is cognitively meaningful. While we consider it important to report these null findings, especially in light of the current debate on ToM in infancy, and appreciate the authors' discussion of these null-findings with reference to future research like the ManyBabies project, **the issue of the interpretation of correlations between chance-level task performances could be discussed in some more depth in the manuscript.**

**As mentioned above (see point 9), we acknowledge the current challenge that the lack of replication of many ToM tasks create for testing stability, as reported by a meta-analysis recently published.**

(4) Relatedly, the **chance-level performance in some of the tasks could be discussed in some more depth** – are there any deviations from the original tasks that might explain the non-replication? How do the findings in specific tasks relate to findings in other non-replications? For example, in the interactive helping task, several studies have replicated a significant difference between the false belief condition and the true belief control condition, while not replicating the above-chance choice of box in the false belief condition itself. The authors’ findings seem to be in line with these previous findings.

**Indeed, our results are in line with previous studies. Because the data from Wave 1 are already discussed in publications, we do not emphasize that issue. Our set of tasks includes strict (knowledge inference, anticipatory looking, interactive false belief ) as well as conceptual (VOE FB and Goal Attribution) replications. Some tasks were replicated (Goal attribution, Knowledge Inference, VOE Goal) while others were not (VOE FB, Anticipatory looking). As mentioned in the manuscript, the current state of the art is that most tasks based on spontaneous responses have been challenging to replicate, as reported in a recent meta-analysis (Barone et al. 2019).**

(5) A related issue is the interpretability of null-findings: From the lack of correlations in study 1 and part of study 2, the authors conclude a lack of stability of the tasks that may support a two system model of implicit and explicit ToM. Concerning these null-findings, it is important to note **that absence of evidence is not evidence of absence**. We suggest to rephrase these parts as “lack of evidence for stability/continuity” etc. rather than “lack of stability” etc. and refer to positive evidence for a dissociation as for example found in a recent paper by Grosse Wiesmann and colleagues (2020, PNAS).

**We agree with a more cautious statement and have edited the ms accordingly. We also cite the Grosse Wiesmann paper as showing dissociation at the neural level, in addition to the behavioral dissociation reported in preschoolers. (see p. 40).**

(6) Comments 3 and 5 also make the conclusion of task specificity of the implicit-explicit correlation problematic as this argument builds on the presence of certain correlations but absence of others. We therefore suggest to remove or rephrase this argument.

**This conclusion no longer stands given the lack of significant correlation across the board.**

(7) The authors included power analysis for both studies, however the assumed effect size for all tasks of 0.5 seems rather large. Is this effect size based on prior literature?

**We aimed for a conservative test and maximized sample size, given past literature.**

Minor Comments

(8) We suggest to include some more motivation of the task design in the introduction: why were completely different tasks used in study 2 than 1 and why were exactly these tasks chosen? Why were children in study 1 retested at different time points (4 and 5 years)?

**We explained in the discussion of Study 1 that given the limitations of the VOE task (carry over effects across 2 sessions) a new set of tasks all based on a helping paradigm seem desirable to generalize the findings. As for the reason for testing children at different ages, this was not planned but resulted from testing the 2 samples over a long period in infancy and over 6 months at Wave 2 (see p. 9).**

(9) Some of the tables lack a description including the abbreviations used in the table as well as the correlation method used.

**This has been corrected.**

(10) It wasn’t fully clear to us whether the samples of study 1 and 2 were independent samples or whether the same children were tested with the different tasks of study 1 and 2. We suggest the authors specify this explicitly in the manuscript.

**These are different samples. It is now clearly specified in the manuscript (see p. 23)**

(11) References:

l. 804: We suggest to also add reference to a similar account by Southgate (2020, Psychol Review, preprint available since 2018).

**Thank you for pointing at this reference. It has been added.**
l. 824: "several failed attempts at replicating other versions of the anticipatory looking paradigm have recently been reported in both adults and children (39, 58-60).” Reference 59 does not report a failed replication of anticipatory looking tasks. Instead references 13 and 14 of the same authors do.

**We have corrected this mistake.**
